# A Case Report and Review of Literature on Spinning-Induced Rhabdomyolysis Among First-Time Female Spinners

**DOI:** 10.7759/cureus.85199

**Published:** 2025-06-01

**Authors:** James C George, Brijesh Valsalan, Ramesh Bhaskaran

**Affiliations:** 1 Orthopaedics, Believers Church Medical College, Thiruvalla, IND; 2 Orthopaedics, Aster Hospital, Sharjah, ARE; 3 Internal Medicine, Aster Hospital, Sharjah, ARE

**Keywords:** cycling, exercise-induced muscle damage, rhabdomyolysis, spinning, women’s health

## Abstract

Spinning is an indoor static cycling training program, and spinning-induced rhabdomyolysis (SIR) is a type of exercise-induced rhabdomyolysis causing systemic and local complications. In this case report, we would like to address its relevance to young women, especially first-time or unfit spinners, and the precautions that should be taken. A 24-year-old woman presented with bilateral thigh pain and dark brown-colored urine after a 45-minute first-time spinning session. She had a creatinine phosphokinase level of 108,166 U/L and myoglobinuria. She was diagnosed with SIR, treated with adequate hydration and urine alkalinization, and was discharged without any complications. Even though the reports are quite limited and focused mainly on the treatment, in view of the high incidence noted in a particular group of people, we recommend public awareness and low-intensity training for all young women engaging in their first indoor cycling session to prevent SIR.

## Introduction

Rhabdomyolysis is a condition where there is damage to the skeletal muscles and subsequent release of muscle proteins, leading to local and systemic complications. The most common etiologies include crush injury, burns, and over-exertion syndromes [1]. Elevated creatinine phosphokinase (CPK), lactate dehydrogenase (LDH), transaminases, and myoglobinuria can lead to compartment syndrome and acute kidney injury (AKI).

Spinning is an indoor cycling exercise program often used for weight management and cardiovascular fitness. It involves different levels of cadence, resistance, and duration, with standing and sitting styles. It has become very popular due to the ease of doing it. The quadriceps and gluteus maximus are the main muscles in action, contracting mostly in an eccentric pattern. Therefore, exercise-induced rhabdomyolysis (EIR) is more likely to occur among these individuals.

Although spinning-induced rhabdomyolysis (SIR) is often mentioned in the literature (14 articles), the importance is given to the various complications and treatment modalities. This case report highlights the relevance of SIR in young women, particularly first-time or unconditioned spinners, and discusses preventive measures.

## Case presentation

**Figure 1:**
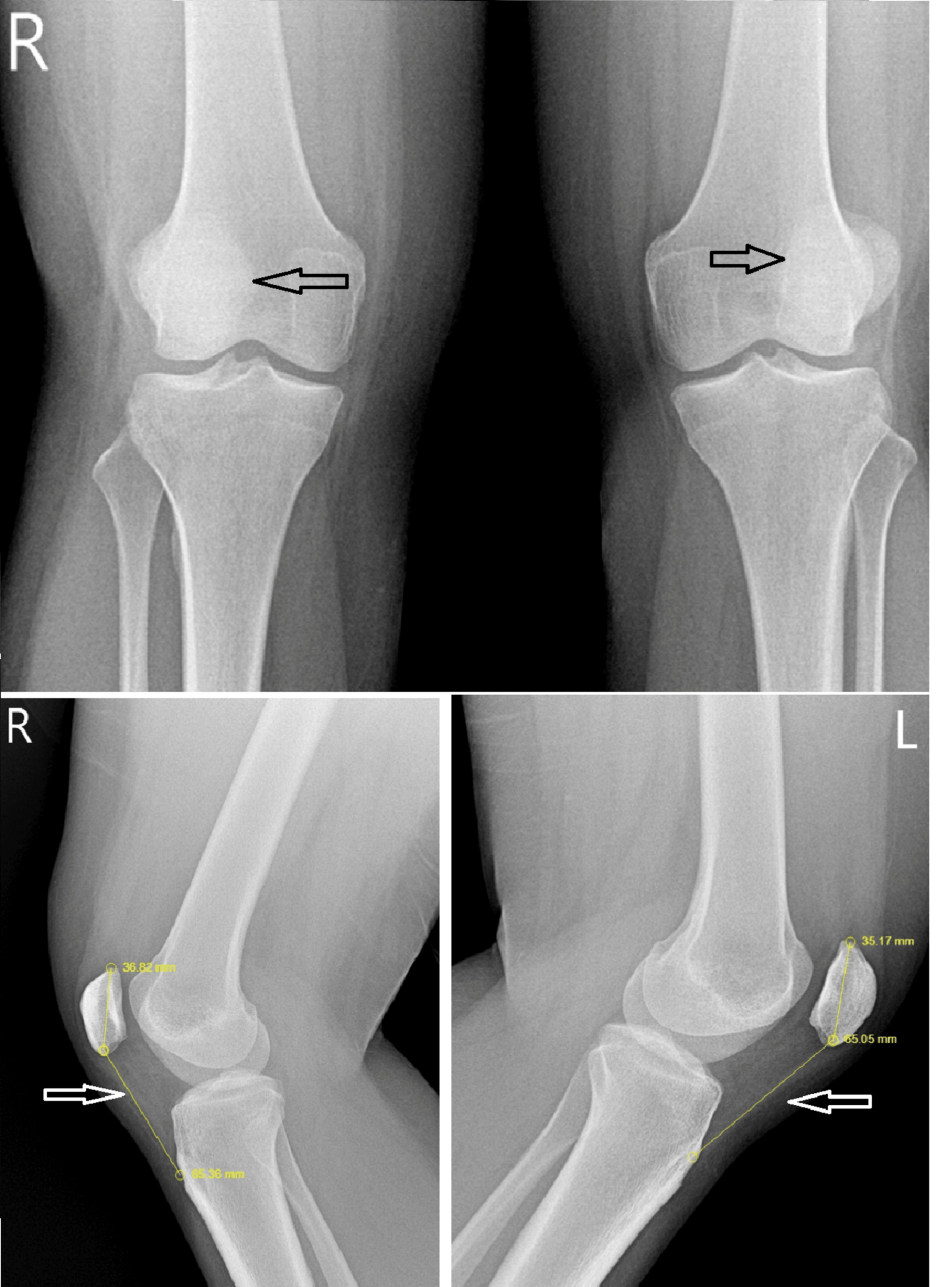
Lateralized patella with an increased Insall-Salvati ratio (1.7), indicative of potential quadriceps dysfunction with patella alta (shown by the arrow).

A 24-year-old woman presented to the outpatient clinic with a history of bilateral thigh pain, difficulty bending her knees, difficulty climbing stairs, and dark brown-colored urine in the morning for the past two days. Seventy-two hours prior to her clinic presentation and 24 hours before the onset of symptoms, she underwent 5 minutes of rigorous standing spinning and 45 minutes of sitting spinning at moderate speed with minimal resistance. This was the first time she was participating in indoor cycling. She did not have any past medical illnesses. On clinical examination, she had tender quadriceps with mild effusion of the knee joints. She had a BMI of 29.6 and a resting heart rate of 116 beats per minute. She was not on any medications for any illness or weight reduction. There was no history of fever or flu-like symptoms prior to the workout. A plain radiograph showed patella alta (Figure 1), and an ultrasonogram showed edematous vastus lateralis bilaterally.

Her biochemical examination results are shown in Table 1. She was hospitalized due to her CPK level of 108,166 U/L. Her creatinine level was normal. Her urine had a pH of 5.5 and became clear on the day of admission. She had a urine myoglobin level of 3334 ng/mL on the day of admission and 1365 ng/mL on the following day. She was admitted to the intensive care unit with continuous ECG monitoring and was hydrated with intravenous and oral fluids, maintaining a urine output of 100 mL/h. Sodium bicarbonate 600 mg twice daily was administered for five days to alkalinize the urine. Hypotension and nephrotoxic drugs were avoided. Her electrolytes were well-balanced, except for a single instance of mild hypocalcemia on the day of admission. She was discharged on day 5 without any systemic or local complications.

**Table 1:** Biochemical parameters during hospitalization CPK, creatinine phosphokinase; LDH, lactate dehydrogenase; AST, aspartate aminotransferase; ALT, alanine aminotransferase; CRP, C-reactive protein.

Investigations	Normal reference range	Day 1 (admission)	Day 2	Day 3	Day 4 (discharged)	Day 7 (outpatient)
CPK (U/L)	<170	108,169	47,699	16,103	7453	1880
LDH (U/L)	135-214	1729	Not done	461	310	240
AST (U/L)	8-33	1830	1349	600	333	114
ALT (U/L)	<33	374	397	334	Not done	220
CRP (mg/dL)	<6	12.70	9.43	Not done	3.72	Not done
Creatinine (mg/dL)	0.6-1.2	0.43	0.55	0.51	0.45	0.59
Calcium (mg/dL)	8.5-10.2	8.22	9.09	8.59	Not done	9.23
Sodium (mmol/L)	135-145	137	135	138	141	141
Potassium (mmol/L)	3.5-5.5	3.98	3.90	3.82	3.89	4.72

## Discussion

EIR is caused by the depletion of adenosine triphosphate (ATP), the cellular source of energy. ATP depletion leads to dysfunction of the Na+/K+-ATPase and Ca²⁺-ATPase pumps, which are essential for maintaining the integrity of myocytes. This depletion causes myocyte injury and the release of intracellular muscle constituents [2]. Myoglobin, which is released from damaged muscles, contains heme, which is nephrotoxic. It can form casts in acidic urine and cause vasoconstriction, leading to AKI.

The reported incidence of EIR is higher in men than in women. This lower incidence is well-documented among female athletes, female firefighters, female marathon runners, female soldiers, and postmenopausal women on hormone replacement therapy. Estrogen is thought to confer a protective effect against exertional rhabdomyolysis in women [3]. The high incidence of EIR noted among the less-trained athletes [4], soldiers performing sedentary duties [5], and less-experienced weight lifters [6] indicated a positive association of lack of fitness and rhabdomyolysis. People with high BMI tend to have more muscle mass, and therefore, BMI cannot be considered an independent risk factor for EIR. The muscle mass of men being more compared to women is also another reason for the high incidence of EIR among men in other training programs like cross-fit, marathon, triathlon, and weightlifting.

Contrary to this, SIR is more commonly reported among women. In a systematic review of 97 patients with SIR, the majority were young women after their first spinning session [7]. In a comparative study involving SIR and other EIR, among the 23 patients, 17 were women with an average age of 27 years, of whom 16 were first-time spinners [8]. Another article on SIR showed 11 young women who presented with dark urine and muscle soreness after their first spinning session [9]. Among the 13 patients reported by Kim, 12 were women under 35 years and first-time spinners [10].

Physical fitness, duration, intensity, and the type of exercise all influence the amount of muscle injury. It is noted that people with poor fitness are more prone to EIR [4]. Eccentric muscle contraction is also a contributing factor for rhabdomyolysis, compared to concentric contraction [11]. Prolonged exertion can lead to dehydration, hypotension, and electrolyte imbalances (hypokalemia and hyponatremia), worsening the situation. Loss of body fluid during exercise of over 2% of body mass can lead to hypotension. A pre-loading of 500 mL before strenuous exercise, with in-between hydration replenishing the fluid loss, is recommended to avoid EIR [12]. In a systematic review of 28 trials on long-duration cycling, hypotonic electrolyte sports drinks were recommended as best for fluid nourishment [13]. Warm-ups before and even the day before performing long-endurance training, gradual increase in exercise intensity, and avoiding hot and humid environments were also found to reduce the incidence of rhabdomyolysis [14].

Considering all these factors, along with the bulk of muscles involved in spinning, especially the quadriceps and gluteus maximus, long-duration spinning in young women during the early phase of training without adequate hydration and warm-ups can lead to significant rhabdomyolysis. While SIR leading to complications such as AKI and compartment syndrome has been reported, the incidence is fortunately low [7,15]. Most patients are discharged within five days after treatment with adequate hydration and urine alkalinization.

Young women, especially those with a high BMI, tend to have adequate energy and muscle bulk. Spinning, being an easy exercise to perform, is often a first-choice activity for weight reduction programs. This endurance and some over-enthusiasm can lead them to spin for prolonged durations at varying intensities. Combined with dehydration, lack of fitness and the muscle mass of the quadriceps make them more prone to rhabdomyolysis after their first spinning session.

## Conclusions

Therefore, we recommend gradual, low-intensity training or periodic training with adequate hydration and replenishing the fluid loss (as determined by the difference between pre- and post-exercise body weight measurements) for all young women engaging in indoor cycling for weight reduction in the early phases of their fitness program. Awareness regarding warm-ups and cool-downs should be given to all beginners involved in indoor spinning. The high incidence of SIR in this demographic warrants increased public health awareness to mitigate its occurrence and potential complications. Making conclusions based on a single case report has its limitations, and therefore, we recommend the need for more studies looking into the incidence and prevention of SIR among young unconditioned first-time female spinners. Patella alta and its contribution to quadriceps overuse, strain, or altered eccentric contraction is also another area for future investigation in SIR.
